# Serum *miR-181d-5p* levels in response to controlled ovarian stimulation: predictive value and biological function

**DOI:** 10.5935/1518-0557.20220053

**Published:** 2023

**Authors:** Maria Gabriela F. Mulato, Luana Marianni Hercolin, Daniela P. A. F. Braga, Amanda S. Setti, Rita de Cassia S. Figueira, Lais Rosa Viana, Carla de Moraes Salgado, Maria Cristina Cintra Gomes Marcondes, Edson Borges Jr., Murilo Vieira Geraldo

**Affiliations:** 1 Department of Structural and Functional Biology, Institute of Biology, University of Campinas - UNICAMP, Campinas, Brazil; 2 Fertility Medical Group, Sao Paulo, Brazil; 3 Sapientiae Institute - Centro de Estudos e Pesquisa em Reprodução Assistida, Sao Paulo, Brazil

**Keywords:** high responder, ovarian stimulation, microRNA, biomarker

## Abstract

The response to controlled ovarian stimulation (COS) for in vitro fertilization (IVF) varies dramatically from one patient to another, affecting success rates. A previous large-scale study identified increased serum *miR-181d-5p* levels in patients with high response to COS prior to stimulation. We aim to evaluate whether the expression of *miR-181d-5p* differs according to the ovarian response to COS in women undergoing intracytoplasmic sperm injection (ICSI) cycles. Samples collected prior to COS for ICSI were split into three groups depending on the ovarian response to COS: poor response (PR), <4 oocytes retrieved (n=25); normal response (NR), ≥8 and ≤12 oocytes retrieved (n=21); and high response (HR), >25 oocytes retrieved (n=20). *miR-181d-5p* serum levels were compared among experimental groups. *miR-181d-5p* levels were increased in the HR group when compared to the PR (*p*=0.0001) and NR groups (*p*=0.0079). *miR-181d-5p* levels correlated with the number of aspirated follicles (*p*<0.0001), retrieved oocytes (*p*<0.0001), and mature oocytes (*p*=0.0002). Increased *miR-181d-5p* levels independently predict a high response (*p*=0.006), with Positive and Negative Predictive Values of 66.7% and 69.4%, respectively. *miR-181d-5p* was also detected in the ovarian tissue in a mouse model. Moreover, computational analysis of *miR-181d-5p* predicted targets and promoter region suggested that this miRNA might be involved in the regulation of key signaling pathways and biological processes for female reproductive biology. In conclusion, *miR-181d-5p* is a promising circulating predictor of high stimulation and potential mediator of the hypothalamus-pituitary-gonad axis, providing opportunities for the individualization of COS protocols.

## INTRODUCTION

Since the advent of assisted reproductive technologies (ART), the number of couples undergoing infertility treatments has increased every year (Faddy *et al*., 2018). According to recent data published by the International Committee Monitoring Assisted Reproductive Technologies (ICMART), from 2008 to 2010, more than four million reproduction cycles have been performed, while more than 1,600,000 cycles have taken place in 2011 (Adamson *et al*., 2018; Dyer *et al*., 2016).

The response to controlled ovarian stimulation (COS), for *in vitro* fertilization (IVF), varies dramatically from one patient to another, affecting success rates and the health of patients (Patrizio *et al*., 2007). The high response to COS may precede the development of ovarian hyperstimulation syndrome (OHSS) (Nyboe Andersen *et al*., 2017), a condition with a low risk of mortality, but which generates important health complications (Boothroyd *et al*., 2015). In order to mitigate the risk of developing OHSS and maintain patient safety, a hyper response to COS usually leads to cancellation of the cycle (Evans *et al*., 2014).

On the other hand, a poor response to the COS protocol may result in insufficient embryos for transfer. The cycle may be cancelled when less than three follicles develop, generating frustration in patients and extra expense through further treatments (Ferraretti *et al*., 2011). According to the European Society of Human Reproduction and Embryology (ESHRE), a poor ovarian response is defined by the collection of fewer than four oocytes in response to a COS protocol of at least 150 IU FSH per day (Ferraretti *et al*., 2011). The management of patients with a poor ovarian response to exogenous gonadotropin stimulation has challenged reproductive specialists for decades. The need to attain an optimal oocyte yield has been recognized, while minimizing the risk of an excessive response and OHSS (Nyboe Andersen *et al*., 2017).

Predicting ovarian response to COS is currently based on different factors, such as age, serum FSH, serum anti-Müllerian hormone (AMH), basal antral follicle count (AFC), and presence of polycystic ovarian syndrome (PCOS). However, there is not a consensus regarding which factors should be taken into consideration when determining the dose of gonadotropin to be administered, and accurate prediction of the ovarian response to COS remains a difficult task (Corbett *et al.,* 2014; Lensen *et al*., 2018; van Tilborg *et al*., 2017). It is clear that new molecular markers for the response to COS are crucial for the development of individualized stimulation protocols.

MicroRNAs (miRNAs) are endogenous, evolutionarily conserved, single-strand non-coding RNA molecules of ~22 nucleotides, which are potent regulators of post-transcriptional gene expression in eukaryotes (Bartel, 2018). The identification of circulating miRNAs has propelled studies focused on the use of miRNAs as biomarkers, since they are detected in various body fluids (Wang *et al*., 2010; Weber *et al*., 2010). MiRNAs are actively secreted into the bloodstream, regulating the gene expression of distant organs in the body, and showing a high predictive value in diseases, such as cancer, pre-eclampsia and PCOS (Jairajpuri *et al*., 2017; He *et al.,* 2015; Roth *et al*., 2014; Sang *et al*., 2013).

Circulating miRNAs have been implicated in ovarian function and ART, and have been detected in follicular fluid and blood plasma of human and animal models (De Bem *et al*., 2017; Freis *et al*., 2017; Santonocito *et al*., 2014; Noferesti *et al*., 2015; Scalici *et al*., 2016). Previously, we used a large scale miRNA expression platform to identify miRNAs that could be used as biomarkers of the response to COS in serum of patients to be submitted to ART (Borges Jr. *et al*., 2020). In the given study, high levels of *miR-181d-5p* were observed in the group of patients with a high response to COS. Bioinformatic analysis indicated that this miRNA may modulate key processes in female reproductive physiology, emerging as a promising candidate for a more detailed analysis. Therefore, in the present study we evaluated *miR-181d-5p* circulating levels in women undergoing intracytoplasmic sperm injection (ISCI) cycles and its correlation with clinical aspects. Moreover, we evaluated the role of the ovaries in the secretion and maintenance of serum *miR-181d-5p* levels in an ovariectomy mouse model.

## MATERIALS AND METHODS

### Experimental design

Sixty-six serum samples were collected from patients prior to COS for intracytoplasmic sperm injection (ICSI), in the private university-affiliated IVF centre Fertility Medical Group, between Jan 2017 and Jan 2018. The participants were <38 years old, and were undergoing their first or second ICSI cycle using fresh oocytes and/or embryos. Samples were retrospectively split into three groups depending on the ovarian response to COS: poor response (PR), <4 oocytes retrieved (n=25), normo response (NR), ≥ 8 and ≤ 12 oocytes retrieved (n=21) and high response (HR), >25 oocytes recovered (n=20). Serum levels of *miR-181d-5p* were measured and compared among experimental groups. In addition, clinical data regarding the treatment was collected, such as the number of aspirated follicles, the number of retrieved oocytes, the number of mature oocytes, maternal age, body mass index (BMI), FSH dose and the level of estradiol on the trigger day.

The study was approved by the Ethics Committee on Research (Human Beings) of the University of Campinas, São Paulo, Brazil, and written informed consent was obtained from all participants (#CAAE: 59443316.6.1001.5404).

### Sample collection

Serum samples were obtained through venipuncture, on the first day of menstrual cycle, prior to beginning COS. After completely filling a serum separation tube, the blood was carefully mixed with a coagulation activating agent and left to stand for 60 minutes. After coagulation, samples were centrifuged at 1500-2000 x g for 10 minutes to separate the serum. Using a Pasteur pipette, the serum was transferred to another tube and stored at -20°C.

### Controlled ovarian stimulation in women and laboratory procedures

Controlled ovarian stimulation was performed using recombinant follicle-stimulating hormone (r-FSH, Gonal-F^®^; Serono, Geneva, Switzerland), with pituitary blockage using a gonadotropin-releasing hormone (GnRH) antagonist, cetrorelix acetate (Cetrotide^®^; Merck KGaA, Serono, Geneva, Switzerland).

Follicular growth was monitored using transvaginal ultrasound examination starting on day 4 of gonadotropin administration. When adequate follicular growth and serum estradiol levels were observed, leuprolide acetate (Lupron^®^; TAP Pharmaceuticals, North Chicago, IL, United States) or recombinant hCG (Ovidrel; Serono, Geneva, Switzerland) was administered to trigger the final follicular maturation. Estradiol levels were measured on the trigger day. The follicle aspiration was scheduled when the largest follicle of the cohort achieved 16 mm in diameter. The oocytes were collected 35 hours later through transvaginal ultrasound ovum pickup. All follicles with diameter larger than 3mM were aspirated. The nuclear status of recovered oocytes was assessed, and those in metaphase II were submitted for ICSI, following routine procedures (Palermo *et al*., 1992).

### Animals and ovariectomy procedures

This study was approved by the Ethics Committee on the Use of Experimental Animals (CEUA/UNICAMP) protocol: 5160-1/2019. Thirteen prepubertal female BALB/cByJ mice from CEMIB - Multidisciplinary Center for Biological Research, UNICAMP were kept in a 12 hours day/night cycle, with free access to water and food. The animals were split into the experimental groups CTR (control group, n=6) and OVA (ovariectomized group, n=7). For the ovary resection in OVA group, 36-days old animals were anesthetized with xylazine chloride 2% (10 mg/kg; König, São Paulo, Brasil) and ketamine hydrochloride 10% (100 mg/kg; Fort Dodge, Iowa, EUA). The ovaries were clipped, removed through dorsal incisions and collected for miRNA analysis, as described below. The animals were followed and medicated for pain throughout the experiment course. Control and OVA groups were euthanized 14 days later with anesthetic overdose of xylazine chloride 2% (30 mg/kg) and ketamine hydrochloride 10% (300 mg/kg). Blood was immediately collected for miRNA analysis, as described below.

### RNA extraction and reverse-transcription quantitative polymerase chain reactions (RT-qPCR)

Small RNAs were extracted from 200 µl of serum samples (human and mouse) using a miRNeasy Serum/Plasma extraction kit (Qiagen, Hilden, Germany), according with the manufacturer’s instructions. Total RNA was extracted from murine ovarian tissue using TRIzol (Thermo-Fisher, Waltham, USA), according with the manufacturer’s instructions. Small and total RNA were quantified with a NanoPhotomer NP80 spectrophotometer (Implen, Munich, Germany). *Caenorhabditis elegans miR-39* (*cel-miR-39*) (miRNeasy Serum/Plasma Spike-in Control; Qiagen, Hilden, Germany) was used for normalisation of gene expression in human or mouse blood samples. In mouse, *RNU6B* was used as endogenous controls for ovarian tissue. cDNA was synthesised with a Taqman MicroRNA Reverse Transcription Kit (Thermo-Fisher, Waltham, USA), following the manufacturer’s instructions. Quantitative PCR (qPCR) for detection of levels of *hsa-miR-181d-5p*, the spike-in and endogenous control was performed by using Taqman^®^ MiRNA Assays (*hsa-miR-181d-5p*, ID Assay 001099; *cel-miR-39*, ID Assay 000200; RNU6B, ID Assay 001093; Thermo-Fisher, Waltham, USA), following the manufacturer’s instructions. All the qPCR reactions were performed in duplicate, at 50^oC^ for 2 min, 95oC for 10 min, and 40 cycles of 95oC for 15 s and 60oC for 1 min, on an ABI7500 thermal cycler (Thermo-Fisher, Waltham, USA). To analyze the data 7500 SDS software was used. The relative levels of *miR-181d-5p* were calculated by the 2-∆∆Ct method. In the human serum analyses, the NR group was used as the calibrator group. In mouse serum or ovarian tissue, the fold changes were plotted against the CTR group. Thus, the mean Ct value for the NR or the CTR group was used as reference for calculation of the relative expression levels for each sample for humans and mice, respectively. As a quality control, the samples that presented Ct values for the normaliser gene (*cel-miR-39* or *RNU6B*) lower or higher than 1.5 Standard deviations were excluded from the analysis.

### Bioinformatics analysis

The analysis of *MIR181C/MIR181D* shared promoter region was performed using a 2kb FASTA sequence upstream from *MIR181C* precursor sequence downloaded using the Genome Browser Platform (https://genome.ucsc.edu). The AliBaba2.1 online software (http://gene-regulation.com/pub/programs/alibaba2/) was used to search for putative binding sites of known transcription factors. The list of putative targets for *miR-181d-5p* was obtained using the miRWalk 2.0 web tool (http://zmf.umm.uni-heidelberg.de/apps/zmf/mirwalk2/). A list of targets predicted by at least eight out of the 12 algorithms used was subsequently submitted to enrichment analysis using the database for annotation, visualisation and integrated discovery (DAVID; https://david.ncifcrf.gov/). Gene interaction network and gene enrichment analysis STRING were generated through the Cytoscape Software (https://cytoscape.org/). The enriched categories using Kegg gene ontology (GO) terms were analysed.

### Statistical analysis

Patient and cycle characteristics, along with clinical and laboratorial results, were analysed using GraphPad Prism version 6.0 (GraphPad Software, San Diego, CA, USA), IBM SPSS Statistics for Windows version 21.0 (IBM Corp., Armonk, NY, USA) and MedCalc version 19.1 (MedCalc Software Ltd, Ostend, Belgium). Variables were tested for normality using the Kolmogrov-Smirnov test. Student’s t-test and the non-parametric Mann-Whitney test were applied for comparison between two independent parametric and non-parametric groups, respectively. One-way analysis of variance (ANOVA) was applied for comparisons between three or more groups with a Gaussian distribution, followed by Bonferroni’s post-hoc test. For non-parametric distributions, the Kruskal-Wallis test was applied, followed by Dunn’s post-hoc test. The correlation between *miR-181d-5p* levels and clinical attributes was calculated by Spearman’s rank correlation coefficient. The positive and negative predictive values of *miR-181d-5p* were assessed by binary logistic regression analysis using the Forward Stepwise method (Wald) and ROC curve (Receiver Operating Characteristic). Values were expressed as mean ± standard deviation, and differences were considered statistically significant when *p*<0.05.

## RESULTS

### Patient and cycle characteristics

Patient and cycle characteristics are summarized in Table 1. As expected, the number of aspirated follicles, retrieved oocytes, and mature oocytes were significantly higher for the HR group, followed by the NR group, while the PR group presented the lowest levels. Increased estradiol levels and oocyte retrieval rates were also observed in the HR group when compared with the PR group (Table 1).

**Table 1:** Characteristics of patients and cycles for the poor, normal and high response groups.

	PR (n=25)	NR (n=21)	HR (n=20)	*p*
Age (years)	32.48±3.51	33.16±2.27	31.92±2.91	0.2180
BMI (kg/m2)	20.87±8.50	23.28±3.34	24.31±3.80	0.9172
FSH dose (IU)	2347.00±774.04	2598.00±558.45	2105.00±570.91	0.1405
Estradiol level (pg/ml)	661.80±1508.20^a^	928.72±917.37^a,b^	2483.84±2810.92^b^	0.0009
Aspirated follicles (n)	4.80±1.15^a^	11.68±1.73^b^	44.64±14.93^c^	< 0.0001
Retrieved oocytes (n)	2.96±0.93^a^	9.44±1.33^b^	36.64±11.34^c^	< 0.0001
Oocyte retrieval rate (%)	65.27±24.21^a^	82.06±13.43^a,b^	82.98±11.49^b^	0.0136
Mature oocytes (n)	2.48±0.96^a^	6.84±1.68^b^	27.92±10.04^c^	< 0.0001
Mature oocyte rate (%)	84.00±19.83	74.29±16.74	76.00±13.68	0.0720

PR, poor response; NR, normal response; HR, high response; BMI, body mass index; Oocyte retrieval rate (number of MII oocytes/number of retrieved oocytes); a ≠ b ≠ c (Kruskal-Wallis test followed by Dunn’s post-hoc test, *p*<0.05).

### *miR-181d-5p* as a predictor of high response to COS

**Figure 1:**
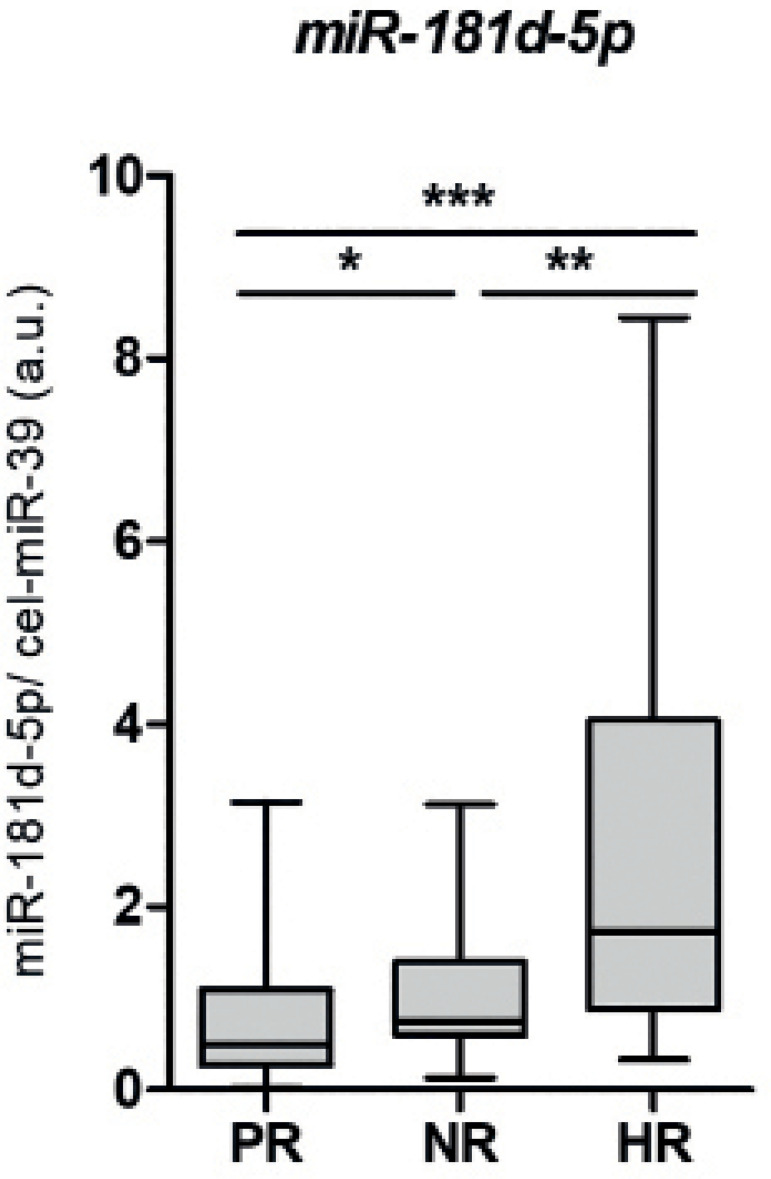
*miR-181d-5p* levels are increased in serum from women with high response to COS. Small RNA fractions were extracted from serum collected from women prior to COS, and were used for cDNA synthesis. *cel-miR-39* was employed as a spike-in for normalization of *miR-181d-5p* expression values. PR, poor response, n=25; NR, normal response, n=21; HR, high response, n=20; *, *p*<0.05; **, *p*<0.01; ***, *p*<0.001. Each plot represents mean ± standard deviation.

As shown in Figure 1, the mean fold change in *miR-181d-5p* levels in the HR group (2.420±2.005) was significantly higher than for the NR (1.164±0.845; *p*=0.0079) group. Additionally, the *miR-181d-5p* levels were significantly higher in the HR and NR groups in comparison to PR group (*p*=0.0411, *p*=0.0001, respectively).

**Figure 2:**
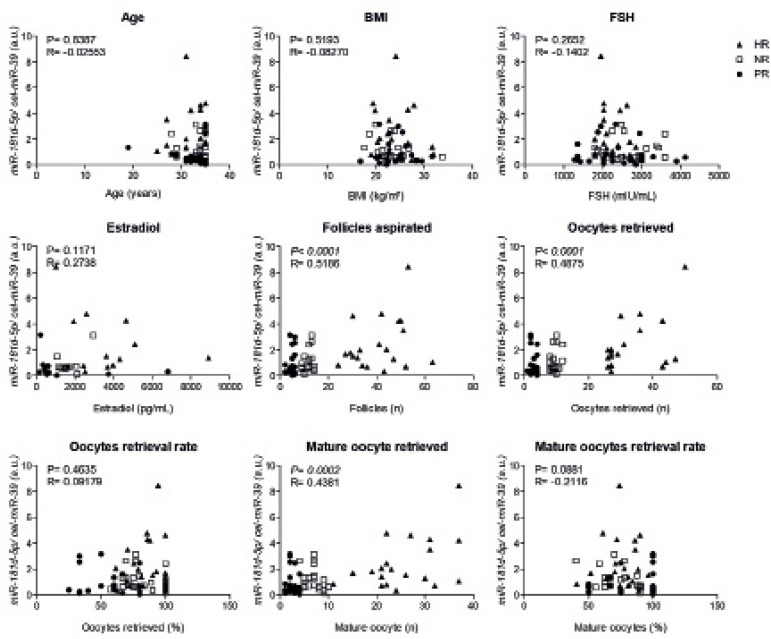
Circulating *miR-181d-5p* levels correlate with clinical outcomes of COS. Serum levels of *miR-181d-5p* were correlated with clinical attributes of the cohort. Spearman’s rank correlation coefficient was calculated. BMI, body mass index; Follicles, follicles aspirated; FSH, follicle-stimulating hormone; PR, poor response; NR, normal response; HR, high response.

Serum levels of *miR-181d-5p* were positively correlated with the number of aspirated follicles (*p*<0.0001, R=0.5186), retrieved oocytes (*p*<0.0001, R=0.4875) and mature oocytes (*p*=0.0002, R=0.4381; Figure 2).

**Figure 3:**
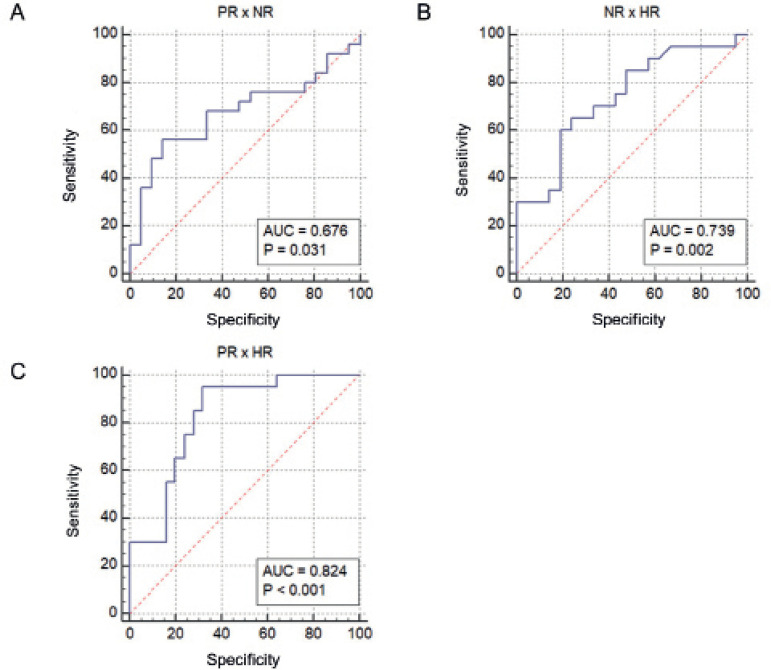
ROC curve of the serum levels of *miR-181d-5p* in the distinction between groups of response to EOC. (a) ROC curve of the PR x NR group ratio, showing AUC=0.676, *p*=0.031, standard error=0.0819, sensitivity=56, specificity, 85.71, cutoff ≤0.572. (b) ROC curve of the HR x NR group relationship, showing AUC=0.739, *p*=0.002, standard error=0.0079, sensitivity=65, specificity=76.19, cutoff> 1.327. (c) ROC curve of the PR x HR group ratio, showing AUC=0.824, *p*<0.001, standard error=0.063, sensitivity=95, specificity=68, cutoff>0.675. (NR, normal response; PR, poor response; HR, high response; AUC, area under the curve).

ROC curve and logistic regression analysis were performed to assess the potential of *miR-181d-5p* levels in discriminating HR and PR from the normal response (NR group) (Figure 3). Our results indicate that circulating *miR-181d-5p* levels predict higher (HR group) from normal response (NR group) (*p*=0.002; AUC=0.739; Figure 3b), independently of age and FSH levels, with positive (PPV) and negative (NPV) predictive values of 69.2% and 69.4%, respectively, and sensitivity and specificity of 45% and 86.2%, respectively (Table 2). Also, *miR-181d-5p* levels discriminate HR group from PR (AUC=0.824, *p*<0.001) (Figure 3c), with PPV=73.3% and NPV=70% (Table 2). Despite a significant *p*-value was observed in the ROC curve (*p*=0.031; AUC=0.676, Figure 3a), the logistic regression analysis showed that *miR-181d-5p* levels could not predict PR from NR (*p*=0.267; Table 2).

**Table 2:** *miR-181d-5p* predicts hyper response to COS independently of age and FSH levels. Binary logistic regression was applied to evaluate the influence of *miR-181d-5p* levels on prediction of COS response.

Groups	Variable	p-value	Exp(B)	Sens.	Spec.	PPV	NPV
PR *vs*. HR	*miR-181d-5p*	0.008	2.351	55.0%	84.0%	73.3%	70.0%
	age	0.275	(n.p.)				
	FSH	0.929	(n.p.)				
NR *vs*. HR	*miR-181d-5p*	0.006	2.483	42.1%	86.2%	66.7%	69.4%
	age	0.062	(n.p.)				
	FSH	0.147	(n.p.)				
PR *vs*. NR	*miR-181d-5p*	0.267	(n.p.)	n.a.	n.a.	n.a.	n.a.
	age	0.392	(n.p.)				
	FSH	0.155	(n.p.)				

n.p., not present in equation; n.a., not applicable; Sens., sensitivity; Spec., specificity; PPV, positive predictive value; NPV, negative predictive value; FSH, follicle-stimulating hormone.

### The ovaries as a source of *miR-181d-5p*

**Figure 4:**
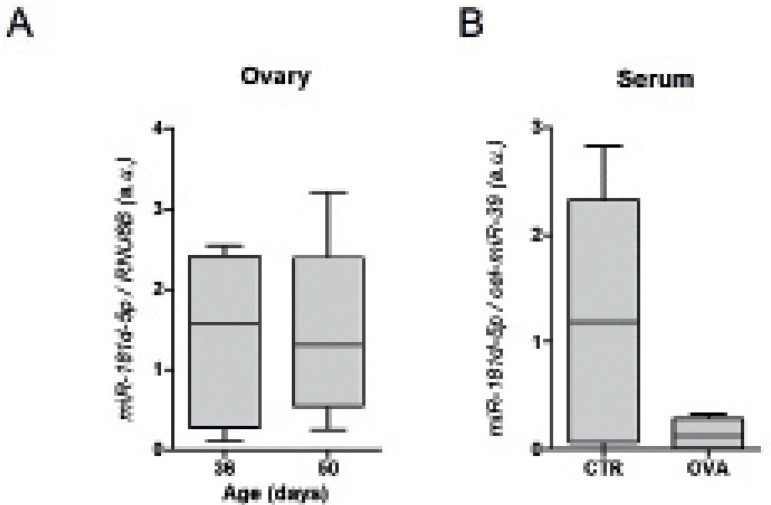
*miR-181d-5p* expression in murine ovarian tissue. (a) Total RNA was extracted from the ovaries removed from ovariectomized animals (31 days-old) and from the ovaries removed from female mice from CTR group at the end of the experiment (50 days-old). *RNU6B* was used as an endogenous control. (b) Small RNA was extracted from blood collected from the ovariectomized (OVA) and the control group (CTR) animals. Spiked-in *cel-miR-39* was used for the normalization of the serum miRNA quantification.

We questioned whether the ovarian tissue could be the main source of circulating *miR-181d-5p*. Mature *miR-181d-5p* could be detected in ovarian tissue from 36- and 50-days old BALB/cByJ animals (Figure 4a). We then submitted prepubertal female mice to ovariectomy and evaluated *miR-181d-5p* serum levels. A decrease of 62% in median levels of *miR-181d-5p* was observed in ovariectomized animals in comparison with the control group (Figure 4b). However no statistical significance was found, probably due to the variance observed in the control group.

### *miR-181d-5p* and the female reproductive biology

**Figure 5:**
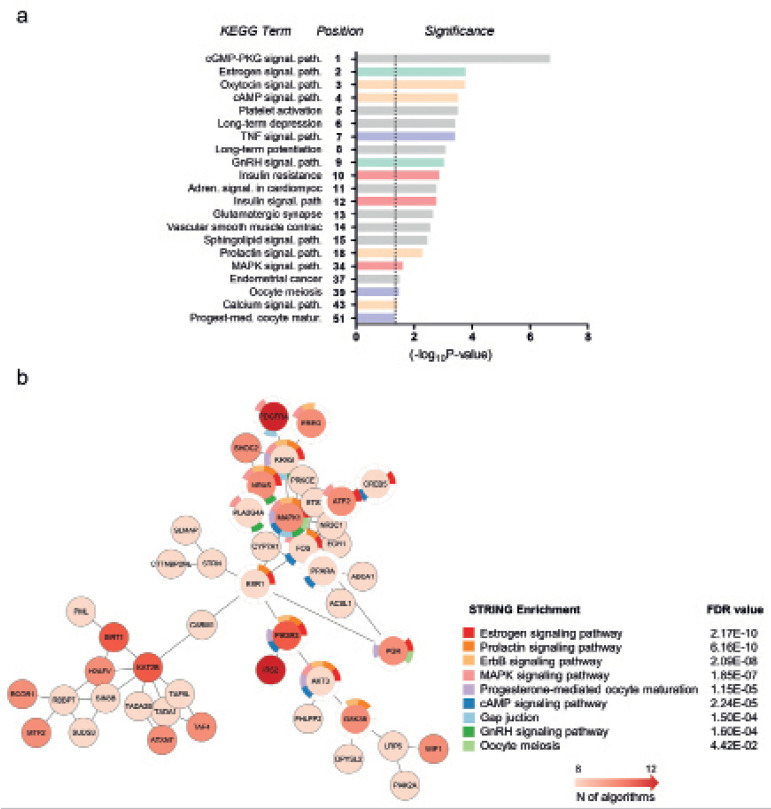
Gene set enrichment analysis of *miR-181-5p* targets. A representative list of the top 15 Kegg GO terms and other relevant terms enriched among *miR-181d-5p* targets. (a) The list of predicted targets of miR-181d-5p was obtained using the miRWalk tool and was subjected to gene set enrichment analysis using the DAVID annotation tool. The enriched Kegg GO terms are shown to the left side of the graph. The bars represent -log10 p-values and the dashed line indicates statistical significance (*p*=0.05). The position of the Kegg GO terms on the list of enriched processes is shown on the Y axis. (b) To construct the gene interaction network with gene enrichment of *miR-181d-5p*, we used targets that were predicted by at least 8 from 12 algorithms, with no additional interactions and with a confidence interval of 0.95.

In order to understand the potential role of *miR-181d-5p* in the female reproductive biology, we used bioinformatics tools to identify the putative network modulated by this miRNA. The analysis of *miR-181d-5p* predicted targets indicates that this miRNA may modulate crucial signaling pathways in female reproductive biology, such as estrogen, cAMP, oxytocin, prolactin and GnRH pathways (Figure 5a and b). Moreover, key members of different GO Terms related with female reproductive biology, such as the “oocyte meiosis” and “progesterone-mediated maturation” are potentially targeted by *miR-181d-5p*.

**Figure 6:**
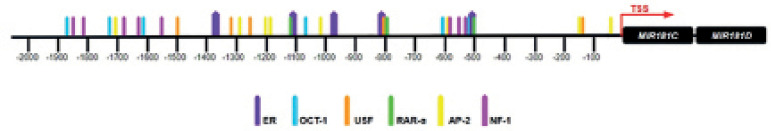
Promoter region of *miR-181d-5p* harbors putative binding sites for transcription factors involved in key signaling pathways for female reproductive biology. A sequence harboring 2kb upstream from the *MIR181C*/*MIR181D* shared promoter region was downloaded using the Genome Browser. The AliBaba2.1 online software was used to search for putative binding sites of known transcription factors.

In addition, the analysis of the promoter region of the *MIR181C/MIR181D* cluster using Alibaba2 bioinformatic tool revealed putative binding sites for key transcription factors for female reproductive biology. For example, we found 5 putative binding sites for ER, suggesting that ER signaling could influence the transcription of this miRNA cluster (Figure 6) during the menstrual cycle.

## DISCUSSION

Clinical and biochemical attributes are routinely used to estimate the dosage of gonadotropin to be used for each patient. To date, AMH levels and AFC, with and without association to age, have been the most commonly used criteria (Ferraretti *et al*., 2011). However, the predictive value of these factors for the individualization of COS protocols are still a matter of debate (Boothroyd *et al*., 2015; Corbett *et al*., 2014; Lensen *et al*., 2018; van Tilborg *et al.,* 2017). Thus, the identification of minimally invasive molecular markers of COS response could be valuable in designing personalized medicine and providing higher success rates. Here we show an association between increased *miR-181d-5p* circulating levels and high response to ovarian stimulation. Circulating levels of *miR-181d-5p* before stimulation correlated with important post-COS clinical features, such as the number of follicles and retrieved oocytes. Notably, binary logistic regression highlighted *miR-181d-5p* as an independent predictor of high response.

It is important to point out that most of the studies focused on quantifying miRNA expression after COS. By contrast, a study by Zhao *et al*. (2015) found that reduced *miR-16* and *miR-223* levels in serum from women with PCOS prior to COS could predict OHSS; however, the role of miRNAs in predicting aberrant COS responses before an IVF cycle remains unclear.

An increasing number of studies have focused on the role of miRNAs in ovarian function. In this context, Kim *et al*. (2013) have shown differential expression of *let-7b* in dominant follicles resulting in mature oocytes, in comparison with those forming immature oocytes. In addition, the use of abnormal miRNA levels in serum is being actively explored in IVF procedures (Kim *et al*., 2013). The analysis of a set of miRNAs, such as *miR-30a, miR-140* and *let-7b*, in follicular fluid was found to be useful in discriminating patients with PCOS from those with normal response to COS (Scalici *et al*., 2016). Moreover, increased *miR-15a-5p* levels were observed in follicular fluid from patients with a poor response after COS (Zhang *et al*., 2017). In addition, Karakaya *et al*. (2015) have shown that increased expression of *miR-21-5p* in cumulus cells is associated with a poor response to COS.

The *miR-181d-5p* is aberrantly expressed in tissue from gliomas (Khalil *et al*., 2016), and in serum from patients with hepatocellular carcinoma (Pascut *et al*., 2019). Interestingly, increased *miR-181d-5p* expression has been described in ovarian and breast cancer (Lee *et al*., 2012; Fahim *et al.,* 2020). Also, *miR-181d-5p* is detected in cancer-associated fibroblasts (CAFs) derived from breast tumors, promoting tumor progression (Wang *et al*., 2020).

The molecular mechanism by which *miR-181d-5p* levels are increased in serum from patients prone to high response to COS and its tissues of origin and destination is still unclear. We detected the expression of *miR-181d-5p* in murine ovaries and reduced circulating levels after ovariectomy. Although the large variance in the control group did not allow statistical significance, this result urges a more detailed investigation to evaluate the ovary as a possible source of this miRNA.

Our bioinformatics analysis revealed that the shared promoter region by the cluster *MIR181C*/*MIR181D* harbours potential binding sites for several transcription factors involved in Estrogen signaling, such as ER, TFAP2A, NR2F2, SP1, GATA2/3 and ETS-1. In fact, a paper from Masuda *et al*. (2012) showed that *miR-181d-5p* is one of the identified up regulated miRNAs in MCF-7 breast cancer cells after exposure to Estrogen. A recent paper from Benedetti *et al*. (2021) describes the intricate regulation network between *miR-181d-5p* and Estrogen signaling cascade. Also, papers by Castellano *et al*. (2009) and Maillot *et al.* (2009) have shown the down-regulation of mir-181 family expression after treatment with Estrogen in breast cancer models. Our bioinformatics analysis indicates that *miR-181d-5p* might impact estrogen signal transduction by targeting *ESR1, FOS, CREB* and different MAPK pathway members. Additionally, *miR-181d-5p* might influence the hypothalamic-pituitary-gonadal (HPG) axis by targeting of *GnRH-r, PRKCA* and *PRKCD*. Also this miRNA could influence oocyte meiosis and maturation, targeting *ADCY1 and 2, PTGS2, IGF1, PGR and AR*, which are involved in many aspects of reproductive biology, including follicle maturation, ovulation, implantation and the establishment of pregnancy (Marei *et al*., 2014; Huang *et al*., 2001; McGee & Hsueh, 2000; Minegishi *et al*., 2000; Inagaki *et al*., 2009). In fact, the paper by Ye *et al.* (2013) describes *miR-181d-5p* as one of several miRNAs up-regulated in primary anterior pituitary cells upon treatment with GnRH in vitro. Moreover, the targeting of *BMPR2*, a key receptor for the action of BMPs, by *miR-181d-5p* could impact FSH-induced steroidogenesis (Otsuka & Shimasaki, 2002), follicle maturation and FSH secretion (Lee *et al*., 2001; Otsuka & Shimasaki, 2002; Shimasaki *et al*., 2004; Lee *et al*., 2007; Nicol *et al*., 2008; Gougeon, 2010; Shi *et al*., 2010; Shimizu *et al*., 2012). This allowed us to hypothesize that *miR-181d-5p* could participate in a feedback loop of regulation of the HPG axis, where its expression influences - and is influenced by - Estrogen signaling.

The AFC and AMH levels have been indicated as preferred methods for predicting ovarian reserve with a varied degree of precision (Polyzos *et al*., 2013; La Marca *et al*., 2010; Lukaszuk *et al*., 2013). However, AMH shows large inter-individual variability, indicating a wide range of ovarian reserve among the healthy population (La Marca *et al*., 2010), whereas the range of values described by AFC do not show the same degree of sensitivity owing to technical limitations, restriction to antral follicles of measurable size, and differences in methodology for counting antral follicles (Arce *et al*., 2013; Broekmans *et al*., 2010). Nevertheless, those biomarkers are still the gold-standard in ovarian reserve evaluation. In the present study, we did not compare the predictive value of *miR-181d-5p* serum levels with AMH and AFC levels. Indeed, our primary goal was to evaluate whether the expression of *miR-181d-5p* would differ between “extremely different” groups in terms of ovarian response. As AMH and AFC still have limited predictive values, often because they are indirect measures of ovarian reserve or have substantial inter-patient variability, the combination with *miR1-18d-5p* would be an interesting approach to more accurately predict the response to COS, rather than replace any other biomarkers. Whether the serum level of *miR-181d-5p* is a better predictor of the COS response than any other biomarker, is still to be elucidated. In this matter, the prospective evaluation of *miR-181d-5p* value in the prediction of the high response may clarify its practical clinical value.

## CONCLUSION

In conclusion, our results showed that *miR-181d-5p* might be a modulator of the hypothalamic-pituitary-gonadal axis, and a potential predictor of the response to COS. Further quantification of *miR-181d-5p* in a larger cohort of samples would validate this miRNA as a molecular marker of COS response. This would allow for the individualization of treatment, increasing its success while decreasing the risks and the physical, emotional and economic burden on patients.
